# Fine-tuning blood culture practices: implementing diagnostic stewardship in cardiothoracic surgery patients

**DOI:** 10.1093/icvts/ivaf005

**Published:** 2025-01-11

**Authors:** Jessica Seidelman, Heather Pena, Brittany A Zwischenberger

**Affiliations:** Duke Center for Antimicrobial Stewardship and Infection Prevention, Durham, NC, USA; Division of Infectious Diseases, Department of Medicine, Duke University, Durham, NC, USA; Department of Surgery, Duke University Medical Center, Durham, NC, USA; Division of Thoracic Surgery, Department of Surgery, Duke University Medical Center, Durham, NC, USA

**Keywords:** blood cultures, diagnostic stewardship, blood culture algorithm

## Abstract

Overusing blood cultures (BCxs) can lead to false positives, unnecessary antibiotics and increased healthcare costs. Despite studies on inpatient BCx algorithms, none have focused on cardiothoracic surgery (CTS) patients, with complex postoperative care and invasive devices. This study aimed to evaluate the impact of a BCx algorithm on BCx event (BCE) rates in CTS step-down units. The study was conducted in three CTS step-down units at Duke University Hospital. The BCx algorithm, based on Seidelman *et al.* (2023), was implemented in June 2023. BCE rates, incidence rate ratios (IRRs) and adverse outcome IRRs were compared between pre- and post-intervention periods using ITS and χ^2^ tests. We analysed 4978 BCE during the study period: 3439 (893 patients) pre-intervention and 1539 (452 patients) post-intervention. BCE rates decreased [IRR = 0.78 (95% confidence interval (CI) 0.74, 0.83, *P*-value< 0.01)] without significant differences in adverse outcomes such as central line-associated bloodstream infection (CLABSI) rates (IRR = 0.6, 95% CI 0.17, 2.30), readmission rates (IRR = 0.99, 95% CI 0.88, 1.12) or in-hospital mortality (IRR = 3.53, 95% CI 0.32, 38.90). Our study supports the beneficial effects of a BCx algorithm, which reduces unnecessary BCxs in CTS patients without compromising patient safety.

## BACKGROUND

Excessive blood culture (BCx) use in healthcare settings is associated with adverse outcomes. Unnecessary BCxs can result in false positives, leading to the administration of unwarranted antibiotic therapy and increased healthcare costs [1–3]. The frequency of contamination in BCxs, often mistaken for true bloodstream infections, adds another layer of complexity to patient management [1]. In addition to the direct clinical implications, unnecessary BCxs can result in prolonged hospital stays and an increased burden on healthcare resources [4].

Recently, the shortage of BD BCx bottles has further highlighted the need for more judicious BCx practices [5]. While several studies have explored the benefits of BCx algorithms, including those by Seidelman *et al.*, Theophanous *et al.* and Fabre *et al.*, a lack of research focused specifically on cardiothoracic surgery (CTS) patients [6–9]. CTS patients represent a unique population due to the complexity of their postoperative care, frequent use of invasive devices and increased risk of infections, necessitating a more tailored approach to BCx practices in this patient group.

### Study purpose

The primary objective of this study was to assess the impact of a BCx algorithm on BCx event (BCE) rates in CTS step-down units. By implementing this algorithm, we aimed to reduce unnecessary BCxs and improve diagnostic stewardship while ensuring that patient safety was not compromised.

## METHODS

### Ethical statement

The study (Pro00109734) was approved by the Duke University Health System Institutional Review Board on 11/22/21. Patient consent was waived as a significant proportion of patients were deceased or no longer cared for by a Duke physician.

### Study setting

This study was conducted at Duke University Hospital, a large academic medical centre. The research focused on three CTS step-down units that care for patients following coronary artery bypass grafting, valve replacements, ventricular assist device placements and heart transplants. Most patients in these units underwent perioperative CTS; the minority of non-CTS patients did introduce variability into the population studied. The units were staffed by resident physicians and advanced practice providers, who manage patient care alongside attending physicians.

### BCx algorithm

The BCx algorithm implemented in this study was based on the model developed by Fabre *et al.* [6] and utilized in previously published studies (Seidelman *et al.* [7]and Theophanous *et al.* [9]). The algorithm guides providers on obtaining BCx for new clinical events or documenting bacteraemia clearance based on the incidence of bacteraemia in specific clinical scenarios. Notably, the algorithm suggestions may differ based on the patient’s immune status and/or clinical setting. The algorithm was introduced in June 2023. We defined inappropriate BCxs as BCxs ordered for clinical scenarios where the algorithm indicated to not draw BCxs. Conversely, we defined appropriate BCxs as BCxs taken for clinical indications where the algorithm supported their use. The pre-intervention period (June 2021 to May 2023) was compared to the intervention period (June 2023 to July 2024). The algorithm standardized the decision-making process for ordering BCxs. It was designed to guide clinicians in evaluating clinical signs, symptoms and risk factors before ordering BCxs, thus optimizing their use.

### Study population

The study included adult patients (≥18 years) admitted to CTS step-down units during the study period. Non-CTS patients were defined as patients not assigned to the CTS service and were included if they were admitted to the CTS step-down units at the time of the BCx. BCEs were recorded when a BCx was ordered. Patients with an absolute neutrophil count of <500 cells/µl and transplant recipients were excluded from the study.

### Statistical plan

Data were analysed with an interrupted time-series analysis using Poisson regression to compare BCE rates before and after the algorithm’s implementation. Adverse outcomes, of readmission rates and mortality were compared between the pre- and post-intervention periods using incidence rate ratios (IRRs). All statistical analyses were performed using SAS version 9.4.

## RESULTS

### BCx event rates

**Figure 1:**
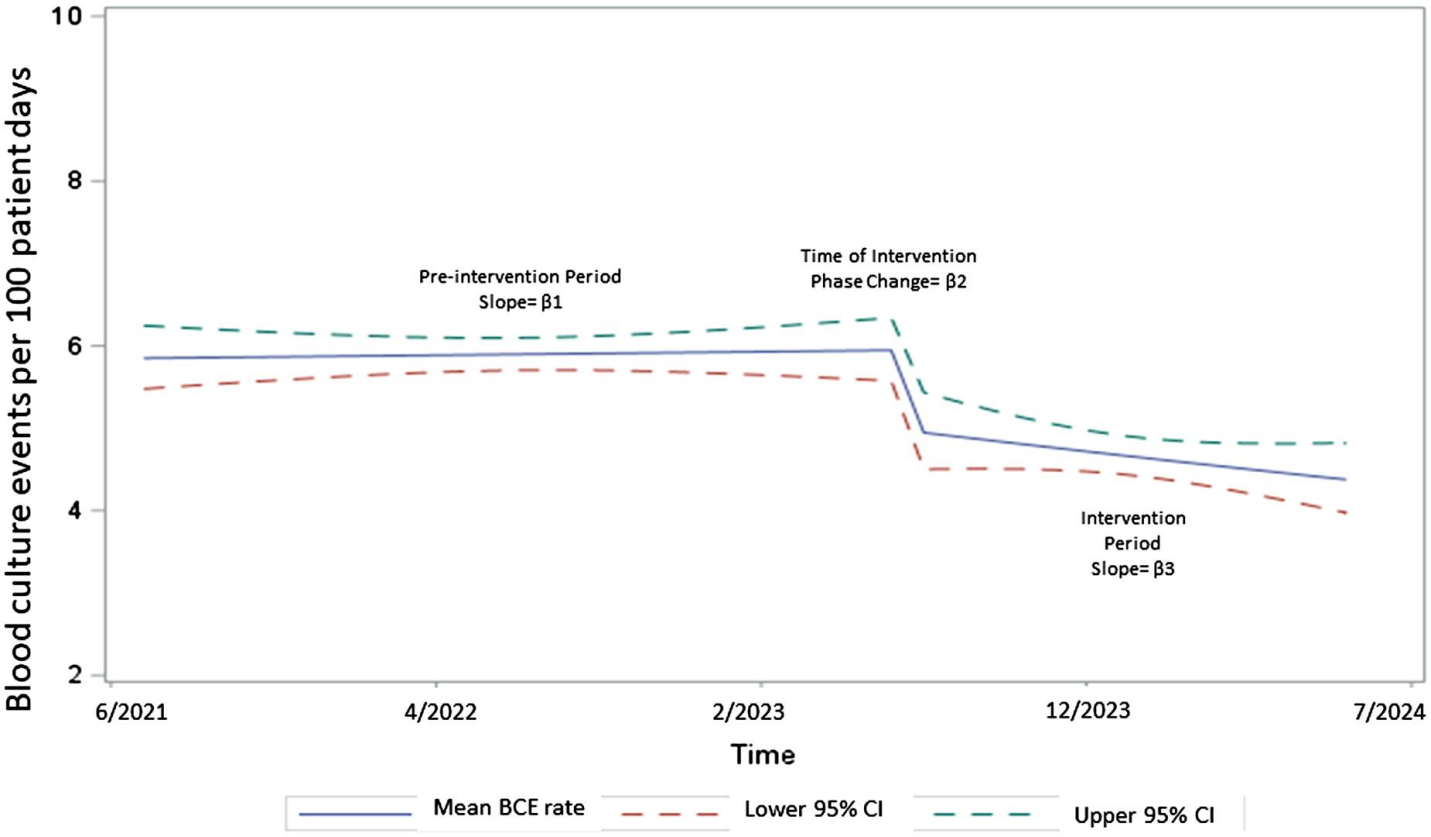
Blood culture event (BCE) rates for the cardiothoracic step-down units in the pre-intervention (June 2021–May 2023) and the post-intervention period (June 2023–July 2024). The pre-intervention period had 3439 in the pre-intervention period over 19 732 patient days (June 2021 to May 2023) and 1539 in the post-intervention period over 10 573 patient days (June 2023 to July 2024). β1 = 0.001 (95% CI −0.004, 0.006, *P*-value 0.77). At the time of the intervention, there was an acute drop measured by the β2 coefficient −0.175 (95% CI −0.298, −0.052, *P*-value <0.01), followed by a slow decrease in slope (β3 = −0.010, 95% CI −0.024, 0.003, *P*-value 0.14).

We analysed a total of 4978 BCE during the study period: 3439 in the pre-intervention period (June 2021 to May 2023) and 1539 in the post-intervention period (June 2023 to July 2024). During the pre-intervention period, there was an insignificant increase in BCE rates of 0.1% (95% confidence interval (CI) −0.004, 0.006, *P* = 0.77) (Fig. 1). During the study period, BCE rates decreased by 17.5% (95% CI −0.298, −0.052, *P* < 0.01). Following the introduction of the algorithm, a further 1.0% decrease was observed (95% CI −0.024, 0.003, *P* = 0.14). The IRR between the pre- and post-intervention phase was 0.78 (95% CI 0.74, 0.83, *P* < 0.01).

### Adverse outcomes

No significant differences were found in the rates of CLABSI, all-cause 30-day readmissions or in-hospital mortality between the pre- and post-intervention periods (Table 1). The pre-intervention CLABSI rate was 0.46 per 1000 line-days, while the post-intervention rate was 0.28 per 1000 line-days (IRR 0.62, 95% CI 0.17, 2.30). Similarly, the 30-day readmission rate was 1.38 per 100 patient days pre-intervention and 1.36 per 100 patient days post-intervention (IRR 0.99, 95% CI 0.88, 1.11). In-hospital mortality remained low, with a rate of 0.00 per 100 patient days pre-intervention and 0.01 per 100 patient days post-intervention (IRR 3.52, 95% CI 0.32, 38.90).

**Table 1:** Comparing patient demographics and adverse outcomes between pre-intervention and post-intervention periods

	Pre-intervention (3439 blood culture events, 893 patients)	Post-intervention (1539 blood culture events, 452 patients)	IRR (95% CI)
Average age in years (std)	59 (15)	58 (16)	
Number of female patients (%)	464 (52)	258 (61)	
Average monthly antibiotic days of therapy (standard deviation)	1768 (255)	1348 (118)	
Central line-associated bloodstream infection rate (CLABSIs/1000 line days)	0.46	0.28	0.62 (0.17, 2.30)
30-day readmission rate (readmission/100 patient days)	1.38	1.36	0.99 (0.88, 1.11)
In-hospital mortality rate (mortality/100 patient days)	0.00	0.01	3.52 (0.32, 38.90)

### Case review data

We reviewed a total of 783 BCE (50.9%) during the post-intervention period. Overall, 569 (72.7%) of BCE were appropriate, 210 (26.8%) were inappropriate and 4 (0.5%) did not have enough data to make an adjudication. One hundred and seventy-eight (22.7%) of the 783 BCE occurred in patients undergoing valve replacement or cardiac bypass surgery, 77 (9.8%) in heart or lung transplant recipients and 209 (36.1%) occurred in non-CTS patients. Of the 210 inappropriate BCE, the most common clinical indication was for isolated fever and/or leucocytosis (130, 61.9%). When these specific BCE were reviewed, 126 (96.9%) resulted in negative cultures and 4 (3.1%) resulted in contaminants. We did not find any BCE for isolated fever and/or leucocytosis that resulted in a true positive culture.

We performed a sub-analysis of the 574 BCE that occurred in only CTS patients. In this subset, 433 (75.4%) of BCE were appropriate, 139 (24.2%) were inappropriate and 4 (0.1%) did not have enough information. The most common reason for inappropriate BCE was again isolated fever/leucocytosis (81, 14.1%); all 81 inappropriate BCE resulted in negative cultures for this group.

## DISCUSSION

### Summary of findings

The findings of this study indicate that the implementation of a BCx algorithm in CTS step-down units can effectively reduce the frequency of BCx orders without adversely affecting patient outcomes. Importantly, no significant differences were observed in key adverse outcomes such as CLABSI rates, readmission rates or in-hospital mortality, suggesting that the reduction in BCx orders did not compromise patient safety. Lastly, we did not find any true bloodstream infections when BCxs were ordered for isolated fever and/or leucocytosis in this population.

### Uniqueness of CTS patients

CTS patients present unique challenges in terms of infection risk and postoperative care. These patients often require invasive monitoring and prolonged hospital stays, which increase their susceptibility to infections. As a result, clinicians may be more inclined to order BCxs as a precautionary measure, even in the absence of clear signs of infection. The primary challenge in implementing the BCx algorithm was shifting the culture around ordering practices and addressing concerns about potential criticism for not ordering cultures. We addressed this barrier by gaining buy-in from the primary surgeons and weekly audit/feedback. The algorithm’s ability to guide clinicians in identifying when BCxs are appropriate is particularly valuable in this population.

### Inclusion of non-CTS patients

The way that the BCx numbers were obtained was based on unit type. However, these units sometimes house non-CTS patients. The providers for these non-CTS patients did not receive direct education on the BCx algorithm, which may have diluted the intervention’s impact. Additionally, the retrospective nature of the study and the reliance on chart reviews may have introduced misclassification bias, further confounding the results.

### Limitations

This study was based on retrospective chart reviews, which are subject to potential misclassification bias. Second, the mixed cohort inclusion of all patients in the CTS step-down units, regardless of whether they were CTS patients, may have biased the results towards the null. We did not educate other physicians on how to use this algorithm and thus BCE occurred in non-CTS patients according to usual care. Third, outcomes comparisons are based on a historical control, which makes it impossible to attribute outcomes to the algorithm alone. Moreover, differences in patient demographics, case complexity, staff experience and resource availability over time may introduce biases, complicating interpretation and potentially confounding intervention effects. Also, we are unable to provide additional demographic data to compare the pre- and post-intervention population based on the limitations of the data report. Finally, the study was conducted at a single academic medical centre, limiting the generalizability of the findings to other institutions.

## CONCLUSION

The implementation of a BCx algorithm in CTS step-down units can reduce BCE rates without compromising patient safety. The findings suggest that diagnostic stewardship efforts can be enhanced by implementing standardized algorithms. Future research should explore the integration of machine learning algorithms to assist clinicians in determining when BCxs are warranted. Additionally, expanding the study to include a larger CTS patient population could help clarify the algorithm’s generalizability and provide power for subgroup analyses.

## Data Availability

Due to patient privacy concerns, access to the raw data is restricted. Researchers interested in accessing the data should contact the corresponding author for approval and a data use agreement.

## Notes

The data contained in this manuscript has never been presented at a meeting
